# Agoraphobic avoidance in patients with psychosis: Severity and response to automated VR therapy in a secondary analysis of a randomised controlled clinical trial

**DOI:** 10.1016/j.schres.2022.10.008

**Published:** 2022-12

**Authors:** Daniel Freeman, Sinéad Lambe, Ushma Galal, Ly-Mee Yu, Thomas Kabir, Ariane Petit, Laina Rosebrock, Robert Dudley, Kate Chapman, Anthony Morrison, Eileen O'Regan, Elizabeth Murphy, Charlotte Aynsworth, Julia Jones, Rosie Powling, Jenna Grabey, Aitor Rovira, Jason Freeman, David M. Clark, Felicity Waite

**Affiliations:** a Department of Psychiatry, University of Oxford, Oxford, UK; b Oxford Health NHS Foundation Trust, Oxford, UK; c Oxford Primary Care Clinical Trials Unit, Nuffield Department of Primary care Health Sciences, University of Oxford, Oxford, UK; d McPin Foundation, London, UK; e Cumbria, Northumberland, Tyne, and Wear NHS Foundation Trust, Newcastle upon Tyne, UK; f Newcastle University, Newcastle upon Tyne, UK; g Avon and Wiltshire Mental Health Partnership (AWP) NHS Trust, Bath, UK; h Greater Manchester Mental Health Foundation Trust, Manchester, UK; i Division of Psychology and Mental Health, University of Manchester, Manchester, UK; j Nottinghamshire Healthcare NHS Foundation Trust, Nottingham, UK; k Department of Experimental Psychology, University of Oxford, Oxford, UK

**Keywords:** Agoraphobia, Paranoia, Hallucinations, Virtual reality (VR), Therapy, Schizophrenia

## Abstract

**Background:**

The social withdrawal of many patients with psychosis can be conceptualised as agoraphobic avoidance due to a range of long-standing fears. We hypothesised that greater severity of agoraphobic avoidance is associated with higher levels of psychiatric symptoms and lower levels of quality of life. We also hypothesised that patients with severe agoraphobic avoidance would experience a range of benefits from an automated virtual reality (VR) therapy that allows them to practise everyday anxiety-provoking situations in simulated environments.

**Methods:**

345 patients with psychosis in a randomised controlled trial were categorised into average, moderate, high, and severe avoidance groups using the Oxford Agoraphobic Avoidance Scale. Associations of agoraphobia severity with symptom and functioning variables, and response over six months to brief automated VR therapy (gameChange), were tested.

**Results:**

Greater severity of agoraphobic avoidance was associated with higher levels of persecutory ideation, auditory hallucinations, depression, hopelessness, and threat cognitions, and lower levels of meaningful activity, quality of life, and perceptions of recovery. Patients with severe agoraphobia showed the greatest benefits with gameChange VR therapy, with significant improvements at end of treatment in agoraphobic avoidance, agoraphobic distress, ideas of reference, persecutory ideation, paranoia worries, recovering quality of life, and perceived recovery, but no significant improvements in depression, suicidal ideation, or health-related quality of life.

**Conclusions:**

Patients with psychosis with severe agoraphobic avoidance, such as being unable to leave the home, have high clinical need. Automated VR therapy can deliver clinical improvement in agoraphobia for these patients, leading to a number of wider benefits.

## 1 Introduction

Even after current pharmacological and psychological treatments, many patients diagnosed with psychosis are left with fears. Fears may concern receiving negative judgements, panicking, and what other people or voices might say or do. There may simply be a perceived inability to cope in ordinary situations. All too often the understandable response by patients is to retreat. Situations from which escape is considered difficult are avoided. Even leaving the home can become challenging. If situations are feared or avoided ‘because of thoughts that escape might be difficult or help might not be available in the event of developing panic-like symptoms or other incapacitating or embarrassing symptoms’ then symptoms of agoraphobia are occurring (APA, 2013). Avoidance in patients with psychosis frequently reaches levels equivalent to a diagnosis of agoraphobia, although in clinical services it is more often viewed as indicative of negative symptoms, such as diminished social motivation or experience of pleasure, or difficulties in general functioning. This agoraphobia perspective on social functioning difficulties of patients with psychosis opens up new treatment possibilities that deserve examination. In this paper we report both on the correlates of this anxious retreat and whether severe avoidance can be successfully reduced with brief psychological therapy delivered using immersive technology.

In a survey of 1800 patients diagnosed with non-affective psychosis, anxious avoidance at agoraphobic levels was present in almost two thirds of patients (Freeman et al., 2019a). An established cut-off on the most widely used scale, the Mobility Inventory for Agoraphobia (Chambless et al., 1985), was used to classify the presence of potential agoraphobia. The inventory, developed in the context of anxiety disorder research, asks about anxious avoidance of 26 situations. Many of the situations, such as theatres, restaurants, museums, auditoriums, airplanes, and boats, are remote from everyday life experiences for many patients diagnosed with psychosis. With the guidance of people with lived experience of psychosis, we developed an anxious avoidance scale more suited to the chronic difficulties often seen in patients with psychosis. Designed using the principles of behavioural avoidance tasks (Himadi et al., 1986), the Oxford Agoraphobic Avoidance Scale (O-AS) (Lambe et al., 2021) asks patients to report whether they would get too anxious to complete each of eight everyday tasks progressing in difficulty from ‘Stand outside your home on your own for 5 minutes’ through ‘Walk down a busy street with someone you know’ and ‘Purchase an item in a local shop from a shop assistant’ to ‘Sit in a café on your own for 10 minutes’. The scale has excellent reliability and validity. The expected score function from item response theory (IRT) models and ROC analysis were used to determine four severity ranges for agoraphobic avoidance that could guide assessment in clinical services: average, moderate, high, and severe.

The Oxford Agoraphobic Avoidance Scale was the primary outcome in a clinical trial testing automated VR cognitive therapy (gameChange) for patients diagnosed with psychosis (Freeman et al., 2022). gameChange is a six-session automated VR therapy targeting agoraphobic avoidance of everyday situations and distress when in those situations. The cognitive perspective underlying the design of gameChange is that unfounded fearful thoughts are the central cause of problematic anxiety (Clark, 1999). Importantly, the fearful thoughts persist because of the use of defence (or safety-seeking) behaviours that block the processing of disconfirmatory evidence (Salkovskis, 1991). For example, people avoid entering feared situations or, when in them, rush to leave early, are vigilant for danger, or avoid eye contact (‘within-situation defences’). The threat cognitions are not updated because the absence of harm is attributed to the use of defences. The treatment implication is that defences must be dropped so that the anxious cognitions can be fully evaluated in the feared situations. In gameChange patients can evaluate fears in six different scenarios: leaving the front door to step into the street, getting onto a bus, and visiting a café, a doctor's surgery, a shop, and a pub (Lambe et al., 2020; Knight et al., 2021). A virtual coach, built into the program, guides the user through the therapy. gameChange was evaluated in a randomised controlled trial with 346 patients with psychosis (Freeman et al., 2022). The VR therapy led to significant reductions in anxious avoidance and distress. However, a moderation analysis showed that gameChange was most efficacious on the primary outcome for approximately the quarter of patients who were in the severe range of agoraphobic avoidance.

In this paper we focus on the potential importance of severe agoraphobic avoidance and its treatment in patients with psychosis. We test if agoraphobic avoidance is a marker of greater clinical severity. The prediction is that greater severity of agoraphobia in patients with psychosis is associated with greater fearful thinking, use of within-situation defences, paranoia, negative voices, depression, and hopelessness, and less meaningful activity, lower quality of life, and lower perceptions of recovery. If greater agoraphobic avoidance reflects higher clinical need then its treatment becomes especially important. We report for the first time, in a post hoc analysis, on the secondary outcomes in the gameChange trial by severity of agoraphobic avoidance. We expected that treating severe agoraphobic avoidance, enabling patients to more readily leave the home, would bring improvements in these other outcomes.

## 2 Methods

### 2.1 Design

The first hypothesis concerning correlates of avoidance was tested using baseline assessments (pre-randomisation) from the gameChange clinical trial. The second hypothesis concerning treatment was tested in using data from all time-points in the trial. This is a follow-up to the primary report of the pre-planned outcomes in the gameChange trial (Freeman et al., 2022). gameChange is a randomised controlled trial with single-blind assessment in two parallel groups: gameChange VR therapy added to standard care, and standard care. Randomisation used a permuted blocks algorithm, with randomly varying block size, stratified by site and service type (in-patient/early intervention/community mental health team). Assessments were conducted at 0 (baseline pre-randomisation), 6 (post treatment), and 26 weeks. Trial assessors were masked to group allocation. If an allocation was revealed then re-masking occurred using another assessor. The trial received approval from an NHS Research Ethics Committee (NHS South Central-Oxford B Research Ethics Committee, ref. 19/SC/0075) and was registered prospectively (ISRCTN17308399).

### 2.2 Participants

The main trial inclusion criteria were (Freeman et al., 2019b): adults aged 16 years or older; attending a National Health Service (NHS) mental health trust for the treatment of psychosis; clinical diagnosis of schizophrenia spectrum psychosis (F20-29) or an affective diagnosis with psychotic symptoms (F31.2, 31.5, 32.3, 33.3) (ICD-10) (WHO, 2004); and having self-reported difficulties going outside the home primarily due to anxiety that they would like treated. The main exclusion criteria were: photosensitive epilepsy; significant visual, auditory, or balance impairment; in forensic settings or Psychiatric Intensive Care Unit (PICU); organic syndrome; primary diagnosis of alcohol or substance disorder or personality disorder; or current active suicidal plans. For the current report a participant needed to have completed the Oxford Agoraphobic Avoidance Scale at the baseline assessment (345 out of 346 trial patients did so).

### 2.3 Assessments

#### 2.3.1 Emotional symptoms and processes

##### 2.3.1.1 Oxford Agoraphobic Avoidance Scale (O-AS) (Lambe et al., 2021)

The O-AS lists eight simple tasks progressing in difficulty. Participants are asked whether they could do the task now or whether they could not because of anxiety (Yes = 0, No = 1), which provides the avoidance score (0–8) (higher scores indicate greater levels of avoidance). In the development of the O-AS, score ranges were also determined, using ROC analysis and the expected test score function from IRT models. The expected test score function estimates the predicted test score given the severity spectrum (theta), and its accuracy is determined by the fit of the IRT model to the data and the correlation between raw total scores and theta scores derived from the model. We used IRT to determine these thresholds because theta scores are considered sample dependent and are assumed to be invariant across groups within the population. Hence, the standardised theta scores were suitable for recommending cut-offs on the expected test scores. The average range was set as zero as the expected score function indicated that most people do not avoid any of the O-AS situations. The moderate range was based on ROC analysis indicating a score of one discriminated patients with anxious avoidance in the context of psychosis from non-clinical control individuals. The expected score function was then used to determine the two highest severity categories. This process resulted in the following four avoidance categories: Average (0), Moderate (1–2), High (3–5), and Severe (6–8). For each of the eight tasks participants are also asked on a 0 (no distress) to 10 (extreme distress) scale how anxious they would feel doing it. These distress scores are summed to provide an overall distress score (0–80) (higher scores indicate greater levels of distress).

##### 2.3.1.2 Patient Health Questionnaire (PHQ-9) (Kroenke et al., 2001)

This scale assesses depressive symptoms over the past two weeks. Each of the nine items is rated on a 0 (not at all) to 3 (nearly every day) scale. Higher scores indicate higher levels of depression.

##### 2.3.1.3 Beck Hopelessness Scale (BHS) (Beck and Steer, 1988)

Twenty statements about feelings of hopelessness in the past week are rated as True or False. Higher scores reflect greater hopelessness.

##### 2.3.1.4 Columbia-Suicide Severity Rating Scale (C-SSRS) (Posner et al., 2011)

This interviewer-rated measure from a semi-structured interview assesses the severity of the highest level of suicidal ideation experienced by the patient over the past month. This is rated on an ordinal 6-point scale from 0 (none) to 5 (suicidal intent with plan).

##### 2.3.1.5 Mobility Inventory for Agoraphobia- Alone subscale (AMI) (Chambless et al., 1985)

The degree of current avoidance because of anxiety is rated on a 1 (never avoid) to 5 (always avoid) scale for each of 26 situations. Higher scores indicate greater anxious avoidance.

##### 2.3.1.6 Oxford Cognitions and Defences Questionnaire (O-CDQ) (Rosebrock et al., 2022)

The O-CDQ comprises three subscales assessing threat cognitions that may contribute to agoraphobia (14 items), anxious avoidance (11 items), and within-situation defence behaviours (8 items). Each item is rated on a scale from 0 (never) to 3 (always). Higher scores on each subscale indicate higher levels of the anxiety-related psychological factor.

#### 2.3.2 Psychotic experiences

##### 2.3.2.1 Revised Green et al. Paranoid Thoughts Scale (R-GPTS) (Freeman et al., 2021)

The R-GPTS comprises an eight-item ideas of reference scale and a 10-item ideas of persecution scale. Each item is rated for the past two weeks on a 5-point (0 to 4) scale. Higher scores indicate greater levels of paranoia.

##### 2.3.2.2 Paranoia Worries Questionnaire (Freeman et al., 2020)

This five-item questionnaire assesses the degree to which an individual has been worrying in the past month about others trying to harm them. Each item is rated on a 0 (none of the time) to 4 (all of the time) scale. Higher scores indicate higher levels of worry with a paranoia content.

##### 2.3.2.3 Negative voices when outside

Five items assess auditory hallucinations that inhibit a person from participation in everyday situations outside the home (e.g. ‘I hear voices that make it difficult to go outside’, ‘As soon as I start thinking about going out, my voices tell me bad things are going to happen’). Each item is rated on a 0 (not at all) to 4 (daily) scale. The Cronbach's alpha for this scale is 0.93 (*n* = 315). Higher scores indicate greater occurrence of voices.

#### 2.3.3 Functioning and quality of life

##### 2.3.3.1 Time budget (Jolley et al., 2006)

This time budget measure, completed during a structured interview, assesses meaningful activity levels over the past week with four time blocks for each day rated from 0 to 4. The rating scale is: 0 = nothing, 1 = predominantly passive activity, 2 = an independent activity requiring some planning and motivation, 3 = several 2-rated activities completely filling a time period or a more complex and demanding, but shorter, activity, 4 = time period filled with a variety of demanding independent activities. Higher scores indicate higher levels of meaningful activity.

##### 2.3.3.2 Recovering Quality of Life (ReQoL) (Keetharuth et al., 2018)

ReQoL is a patient-reported outcome measure developed to assess quality of life for people with mental health conditions. It consists of 20 mental health questions, each rated on a scale from 0 to 4 (‘None of the time’ to ‘Most of the time’). Higher scores indicate better quality of life.

##### 2.3.3.3 EuroQoL (EQ-5D-5L) (Herdman et al., 2011)

The Health Index and Health Today scores were used. The Health Index is calculated from responses to five items assessing mobility, self-care, activities, pain, and anxiety/depression. The index was calculated using the crosswalk method. Higher scores indicate higher quality of life. The Health Today score is provided by a rating on a 0–100 visual analogue scale, with endpoints labelled ‘The best health you can imagine’ (100) and ‘The worst health you can imagine’ (0).

##### 2.3.3.4 Questionnaire about the Process of Recovery (QPR) (Neil et al., 2009; Law et al., 2014)

This is a 15-item questionnaire developed collaboratively by service-user researchers and clinicians assessing current recovery. Items are rated on a five-point scale from 0 (strongly disagree) to 4 (strongly agree). Higher scores indicate greater recovery.

#### 2.3.4 Intervention

The gameChange VR therapy is a virtual-reality application recommended for adults (16+) who are anxious about everyday situations. The treatment is a CE marked Class I Active Medical Device- Z301 (Standalone Software), in conformity with the essential requirements and provisions of the EC Directive 93/42/EEC (Medical Devices). Trial hardware was an HTC Vive Pro headset and Dell G5 15 5590 laptop. The treatment is designed to be delivered in approximately 6 sessions, each involving thirty minutes in VR. During the trial a mental health worker – peer support worker, assistant psychologist, or clinical psychologist – helped the person maximise the learning from the VR programme. Staff deliverers did not need to have previous experience of cognitive therapy. The staff member set up the hardware, briefly introduced the treatment concepts, helped the patient put on the VR headset, and started the programme. The staff member also encouraged the person to apply the learning from VR in the real world via setting of between session tasks. The gameChange VR therapy is a cognitive treatment that aims for patients to relearn safety by testing their fear expectations. The user typically stands and can walk a few paces in the scenarios. Within the VR environments a virtual coach guides the person through the treatment. The coach encourages the dropping of defence (safety-seeking) behaviours, the evaluation of fears, and elicits feedback to tailor the progression of the treatment. When first entering VR, the patient goes into the coach's virtual office and is guided in how to use VR. At the beginning of the first session, the virtual coach explains the rationale behind the treatment, and the participant selects one of the six VR scenarios. Each scenario comprises five levels of difficulty and participants work their way through the tasks in each level. The participant can choose a different scenario in each session or repeat a previous scenario. A full description of the design process and VR therapy is provided in two separate publications (Lambe et al., 2020; Knight et al., 2021).

#### 2.3.5 Analysis

Baseline association tests were conducted with SPSS Version 27.0 (IBM, 2020) and outcome analyses with Stata (SE) version 16.1 SE (StataCorp., 2019). Associations were tested using analysis of variance (ANOVA), with post hoc least significant difference tests. The moderation analyses were conducted using linear regression, modelling the baseline outcome measure, treatment assignment, stratification factors, the moderator (agoraphobic avoidance severity), and an interaction between randomised group and the moderator as a fixed effect. *p* < 0.05 was used as the level of statistical significance for all tests. Results are reported as mean differences between treatment groups together with 95 % confidence intervals. Treatment differences were additionally reported as standardised mean differences (mean group difference divided by the standard deviation of the whole trial group at baseline).

## 3 Results

Socio-demographic and clinical information for the patients, presented by agoraphobic avoidance severity groups, are summarised in Table 1. Across the groups the average age was approximately 40. Most people were single, unemployed, outpatients, and prescribed antipsychotic medication. The group with the lowest level of agoraphobic avoidance had the highest proportions of males and of people in employment. The group with the highest levels of agoraphobic avoidance had the highest levels of people unemployed and prescribed antipsychotic and antidepressant medications.

**Table 1:** Basic socio-demographic and clinical information.

	Avoidance severity ranges			
	Average (*n* = 62)	Moderate (*n* = 95)	High (*n* = 101)	Severe (*n* = 87)
Age (years)				
Mean (SD)	35.7 (9.5)	37.7 (13.4)	36.1 (13.2)	39.0 (12.5)
Gender, n (%)				
Female	10 (16.1 %)	38 (40.0 %)	30 (30.0 %)	32 (36.8 %)
Male	52 (83.9 %)	55 (57.9 %)	69 (69.0 %)	55 (63.2 %)
Other	0	1 (1.1 %)	0	0
Prefer not to say	0	1 (1.1 %)	1 (1.0 %)	0
Marital status, n (%)				
Single	52 (85.2 %)	77 (81.9 %)	77 (77.0 %)	62 (78.4 %)
Married/civil partnership	5 (8.2 %)	6 (6.4 %)	13 (13.0 %)	11 (12.6 %)
Cohabiting	0	4 (4.3 %)	4 (4.0 %)	8 (9.2 %)
Separated	1 (1.6 %)	0	1 (1.0 %)	1 (1.1 %)
Divorced	2 (3.3 %)	4 (4.3 %)	5 (5.0 %)	5 (5.7 %)
Widowed	1 (1.6 %)	3 (3.2 %)	0	0
Ethnicity, n (%)				
White	56 (90.3 %)	83 (87.4 %)	78 (78.0 %)	76 (87.4 %)
Black British	0	1 (1.1 %)	1 (1.0 %)	0
Black African	0	1 (1.1 %)	2 (2.0 %)	0
Black Caribbean	1 (1.6 %)	0	1 (1.0 %)	2 (2.3 %)
Indian	0	2 (2.1 %)	0	0
Black Other	0	1 (1.1 %)	0	0
Chinese	0	0	0	0
Pakistani	1 (1.6 %)	1 (1.1 %)	2 (2.0 %)	2 (2.3 %)
Other	4 (6.5 %)	6 (6.3 %)	16 (16.0 %)	7 (8.0 %)
Service type, n (%)				
Community mental health team	34 (54.8 %)	59 (62.1 %)	58 (57.4 %)	58 (66.7 %)
Early intervention service	27 (43.5 %)	36 (37.9 %)	40 (39.6 %)	29 (33.3 %)
In-patient	1 (1.6 %)	0	3 (3.0 %)	0
Employment, n (%)				
Employed full-time (paid)	8 (14.6 %)	6 (7.1 %)	2 (2.2 %)	3 (3.7 %)
Employed part-time (paid)	3 (5.3 %)	5 (6.0 %)	0	0
Employed full-time (voluntary)	0	0	0	0
Employed part-time (voluntary)	1 (1.8 %)	2 (2.4 %)	2 (2.2 %)	0
Unemployed (on benefits)	35 (61.4 %)	59 (70.2 %)	73 (80.2 %)	67 (82.7 %)
Unemployed (not on benefits)	1 (1.8 %)	4 (4.8 %)	4 (4.4 %)	4 (4.9 %)
Student or in training full-time	1 (1.8 %)	5 (6.0 %)	4 (4.4 %)	1 (1.2 %)
Student or in training part-time	2 (3.5 %)	1 (1.2 %)	1 (1.1 %)	0
Self-employed	3 (5.3 %)	0	0	1 (1.2 %)
Home-maker	1 (1.8 %)	1 (1.1 %)	1 (1.1 %)	0
Carer	1 (1.8 %)	0	0	1 (1.2 %)
Retired	1 (1.8 %)	1 (1.2 %)	2 (2.2 %)	3 (3.7 %)
Missing	0	0	2 (2.2 %)	1 (1.2 %)
Mental health diagnosis, n (%)				
Schizophrenia	28 (45.2 %)	37 (38.9 %)	38 (37.6 %)	35 (40.2 %)
Schizotypal disorder	1 (1.6 %)	1 (1.1 %)	0	1 (1.1 %)
Delusional disorder	1 (1.6 %)	0	2 (2.0 %)	1 (1.1 %)
Brief psychotic disorders	1 (1.6 %)	6 (6.3 %)	6 (6.0 %)	1 (1.1 %)
Schizoaffective disorder	1 (1.6 %)	5 (5.3 %)	14 (13.9 %)	6 (6.9 %)
Other psychotic disorder	1 (1.6 %)	0	3 (3.0 %)	1 (1.1 %)
Unspecified psychosis	25 (40.3 %)	36 (37.9 %)	34 (33.7 %)	26 (29.9 %)
Bipolar disorder with psychotic features	3 (4.8 %)	3 (3.2 %)	0	2 (2.3 %)
Depressive disorders with psychotic features	1 (1.6 %)	6 (6.3 %)	2 (2.0 %)	11 (12.6 %)
Major depressive disorder with psychotic features	0	1 (1.1 %)	2 (2.0 %)	3 (3.4 %)
Medication				
Antipsychotic, Yes, n (%)	55 (88.7 %)	85 (89.5 %)	93 (93.0 %)	83 (95.4 %)
Antipsychotic, No, n (%)	7 (11.3 %)	10 (10.5 %)	7 (7.0 %)	4 (4.6 %)
Chlorpromazine equivalent dose mean (SD)	403.8 (330.9)	330.7 (276.8)	391.7 (350.0)	352.9 (262.4)
Anxiolytic, Yes, n (%)	2 (3.2 %)	8 (8.5 %)	11 (11.0 %)	7 (8.0 %)
Anxiolytic, No, n (%)	60 (96.8 %)	86 (91.5 %)	89 (89.0 %)	80 (92.0 %)
Antidepressant, Yes, n (%)	31 (50.0 %)	52 (54.7 %)	59 (59.0 %)	57 (65.5 %)
Antidepressant, No, n (%)	31 (50.0 %)	43 (45.3 %)	41 (41.0 %)	30 (34.5 %)

The associations of psychiatric symptoms and functioning with avoidance severity group are presented in Table 2. The severity of all problems increased across the avoidance severity groups. There were statistically significant differences in levels of persecutory ideation, paranoia worries, voices, depression, hopelessness, agoraphobia (assessed by the AMI), threat cognitions, avoidance (assessed by the O-CDQ), within-situation defences, meaningful activity, quality of life, and recovery. Group differences were not statistically significant for ideas of reference, total paranoia, and suicidal ideation.

**Table 2:** Agoraphobic avoidance severity level and symptom and functioning scores.

Variable	Avoidance level										
	Average		Moderate		High		Severe				
	n	Mean (SD)	n	Mean (SD)	n	Mean (SD)	n	Mean (SD)	df	F	*p*-value
Psychotic experiences											
Ideas of reference (R-GPTS-A)	57	12.00 (8.84)	88	12.64 (9.39)	90	12.99 (8.42)	83	15.10 (9.97)	3, 314	1.63	0.184
Ideas of persecution (R-GPTS-B)	57	12.93^a^ (11.25)	88	13.77^a^ (12.36)	90	17.28^b^ (12.75)	83	17.86^b^ (14.13)	3, 314	2.81	0.040
Paranoia Total (R-GPTS)	57	24.93 (19.20)	88	26.41 (20.54)	90	30.27 (19.61)	83	32.95 (23.03)	3, 314	2.31	0.077
Paranoia Worries (PWQ)	55	7.56^a^ (5.57)	86	8.55^a,b^ (6.15)	90	9.98^b, c^ (6.54)	82	10.84^c^ (5.98)	3, 309	3.98	0.008
Voices	58	7.21^a^ (6.68)	84	6.86^a^ (6.72)	90	9.83^b^ (7.53)	83	10.39^b^ (7.64)	3, 311	4.93	0.002

Emotional symptoms and processes											
Depression (PHQ-9)	58	14.24^a^ (6.05)	90	13.33^a^ (6.42)	96	14.10^a^ (5.90)	83	16.77^b^ (6.35)	3, 323	4.93	<0.001
Hopelessness (BHS)	55	9.65^a^ (5.41)	85	10.32^a^ (6.02)	89	11.28^a,b^ (6.03)	82	12.96^b^ (5.08)	3, 307	4.66	0.003
Suicidal ideation (C-SSRS)	55	0.67 (1.22)	83	1.01 (1.24)	91	1.04 (1.26)	79	1.18 (1.30)	3, 304	1.80	0.148
Agoraphobia (AMI)	59	2.75^a^ (0.66)	91	2.88^a^ (0.64)	95	3.34^b^ (0.55)	85	3.92^c^ (0.63)	3, 326	57.82	<0.001
Threat cognitions (O-CDQ)	62	15.58^a^ (8.45)	93	16.94^a^ (7.85)	99	20.16^b^ (8.72)	86	23.20^c^ (9.79)	3, 336	12.05	<0.001
Avoidance (O-CDQ)	62	11.77^a^ (6.83)	93	14.49^b^ (7.20)	99	19.18^c^ (5.73)	86	24.09^d^ (7.07)	3, 336	50.90	<0.001
Within-situation defences (O-CDQ)	62	12.08^a^ (4.88)	93	13.38^a^ (5.38)	98	15.84^b^ (4.93)	86	16.67^b^ (5.73)	3, 335	12.66	<0.001

Activity and quality of life											
Meaningful activity (Time budget)	51	56.45^a^ (16.24)	83	54.65^a^ (15.50)	84	51.49^a, b^ (16.91)	74	48.34^b^ (18.78)	3, 288	2.97	0.032
Quality of life (ReQoL)	59	40.38^a^ (11.48)	91	36.36^a,b^ (12.70)	94	34.57^b^ (13.40)	84	28.48^c^ (12.58)	3, 324	11.21	<0.001
Quality of life- Health index (EQ-5D)	61	0.64^a^ (0.22)	95	0.59^a,b^ (0.25)	99	0.54^b^ (0.27)	86	0.43^c^ (0.29)	3, 337	9.13	<0.001
Quality of life- Health today (EQ-5D)	61	56.80^a^ (17.85)	94	55.78^a^ (18.12)	99	50.30^b^ (20.50)	86	47.76^b^ (18.57)	3, 336	4.23	0.006
Recovery (QPR)	62	32.73^a^ (7.55)	94	28.76^b^ (10.44)	100	27.74^b^ (11.27)	86	22.57^c^ (11.17)	3, 338	12.04	<0.001

Note. Significant group differences (p < 0.05) are denoted by differing superscript letters.

Outcome results at 6 weeks (end of treatment) and 26 weeks (follow-up) by avoidance severity groups are presented in Table 3, Table 4. Compared to the individuals with severe agoraphobia in the control condition, the individuals with severe agoraphobia who had VR therapy showed significant improvements at six weeks in agoraphobic avoidance (d = 0.63), agoraphobic distress (d = 0.63), agoraphobic avoidance as assessed by the AMI (d = 0.44), ideas of reference (d = 0.51), persecutory ideation (d = 0.63), overall paranoia (d = 0.63), paranoia worries (d = 0.51), recovering quality of life (d = 0.52), and perceived recovery (d = 0.36), but no significant improvements in depression, suicidal ideation, or quality of life assessed by the EQ-5D. These treatment benefits for the individuals with severe agoraphobia in the VR therapy group, compared to the individuals with severe agoraphobia in the control condition, were maintained at 26 weeks for agoraphobic avoidance (d = 0.79), agoraphobic distress (d = 0.77), agoraphobic avoidance as assessed by the AMI (d = 0.76), persecutory ideation (d = 0.39), overall paranoia (d = 0.38), and recovering quality of life (d = 0.53).

**Table 3:** Treatment effects at end of treatment (6 weeks) by agoraphobic severity group.

	VR + TAU	TAU	Adjusted mean difference [95 % CI]^a^	*P* value for mean difference	Test of Interaction (P value)
	(*N* = 173)	(*N* = 172)			
Oxford Agoraphobic Avoidance Scale (O-AS) – avoidance score					
0: Average avoidance, mean (SD) [n]	0.7 (1.3) [27]	0.5 (1.2) [32]	0.26 [−0.74 to 1.25]	0.611	0.014
1–2: Moderate avoidance, mean (SD) [n]	1.3 (1.7) [53]	1.0 (1.4) [37]	0.08 [−0.75 to 0.91]	0.850	
3–5: High avoidance, mean (SD) [n]	2.2 (1.8) [43]	2.6 (2.3) [48]	−0.34 [−1.14 to 0.47]	0.409	
≥6: Severe avoidance, mean (SD) [n]	3.6 (2.8) [36]	5.3 (2.2) [43]	−1.63 [−2.49 to −0.77]	<0.001	

Oxford Agoraphobic Avoidance Scale (O-AS) – distress score					
0: Average avoidance, mean (SD) [n]	35.5 (19.2) [27]	31.3 (16.3) [32]	2.43 [−5.27 to 10.13]	0.535	0.083
1–2: Moderate avoidance, mean (SD) [n]	37.1 (16.0) [53]	35.5 (17.8) [37]	−2.47 [−8.89 to 3.96]	0.451	
3–5: High avoidance, mean (SD) [n]	44.8 (18.6) [43]	49.2 (17.6) [48]	−3.57 [−9.77 to 2.64]	0.259	
≥6: Severe avoidance, mean (SD) [n]	48.1 (20.1) [36]	61.1 (16.1) [45]	−10.54 [−17.12 to −3.96]	0.002	

Agoraphobia Mobility Inventory (AMI-A)					
0: Average avoidance, mean (SD) [n]	2.5 (0.7) [25]	2.6 (0.7) [31]	0.06 [−0.26 to 0.38]	0.707	0.214
1–2: Moderate avoidance, mean (SD) [n]	2.8 (0.6) [51]	2.5 (0.7) [34]	−0.09 [−0.36 to 0.17]	0.496	
3–5: High avoidance, mean (SD) [n]	3.0 (0.7) [40]	3.0 (0.8) [46]	−0.05 [−0.31 to 0.20]	0.675	
≥6: Severe avoidance, mean (SD) [n]	3.3 (1.0) [35]	3.8 (0.7) [41]	−0.35 [−0.62 to −0.09]	0.010	

Ideas of reference (R-GPTS Part A)					
0: Average avoidance, mean (SD) [n]	9.4 (8.1) [24]	9.6 (7.8) [31]	0.10 [−3.27 to 3.46]	0.955	0.045
1–2: Moderate avoidance, mean (SD) [n]	10.5 (7.7) [47]	8.6 (7.3) [34]	−1.03 [−3.90 to 1.83]	0.478	
3–5: High avoidance, mean (SD) [n]	11.8 (9.3) [37]	10.1 (8.8) [42]	0.71 [−2.11 to 3.54]	0.620	
≥6: Severe avoidance, mean (SD) [n]	10.5 (9.2) [33]	13.8 (8.8) [39]	−4.73 [−7.62 to −1.84]	0.001	

Persecutory ideation (R-GPTS Part B)					
0: Average avoidance, mean (SD) [n]	12.4 (12.1) [24]	9.9 (10.6) [31]	1.53 [−2.91 to 5.97]	0.498	0.001
1–2: Moderate avoidance, mean (SD) [n]	12.7 (11.1) [47]	7.8 (9.9) [34]	−1.14 [−4.92 to 2.65]	0.555	
3–5: High avoidance, mean (SD) [n]	15.8 (12.7) [37]	12.5 (12.7) [42]	1.79 [−1.93 to 5.52]	0.345	
≥6: Severe avoidance, mean (SD) [n]	11.0 (12.0) [33]	17.5 (14.4) [39]	−8.16 [−11.97 to −4.35]	<0.001	

Overall paranoia (R-GPTS total)					
0: Average avoidance, mean (SD) [n]	21.8 (19.4) [24]	19.5 (16.8) [31]	1.59 [−5.45 to 8.63]	0.657	0.002
1–2: Moderate avoidance, mean (SD) [n]	23.2 (17.5) [47]	16.4 (16.4) [34]	−2.44 [−8.44 to 3.56]	0.424	
3–5: High avoidance, mean (SD) [n]	27.6 (20.5) [37]	22.7 (20.2) [42]	2.43 [−3.48 to 8.33]	0.420	
≥6: Severe avoidance, mean (SD) [n]	21.5 (20.4) [33]	31.4 (22.4) [39]	−13.07 [−19.11 to −7.03]	<0.001	

Paranoia Worries Questionnaire (PWQ)					
0: Average avoidance, mean (SD) [n]	7.2 (5.7) [24]	5.7 (4.7) [31]	0.89 [−1.54 to 3.31]	0.472	0.006
1–2: Moderate avoidance, mean (SD) [n]	6.4 (5.4) [45]	5.1 (5.5) [33]	−0.64 [−2.73 to 1.45]	0.547	
3–5: High avoidance, mean (SD) [n]	9.7 (6.4) [38]	8.1 (6.2) [43]	1.73 [−0.24 to 3.70]	0.085	
≥6: Severe avoidance, mean (SD) [n]	7.8 (6.7) [33]	10.3 (6.5) [38]	−3.14 [−5.20 to −1.08]	0.003	

Depression (Patient Health Questionnaire-9)					
0: Average avoidance, mean (SD) [n]	12.0 (6.6) [23]	11.3 (5.4) [32]	0.83 [−1.93 to 3.60]	0.554	0.646
1–2: Moderate avoidance, mean (SD) [n]	11.6 (5.4) [51]	10.3 (5.7) [35]	0.06 [−2.24 to 2.36]	0.960	
3–5: High avoidance, mean (SD) [n]	13.9 (6.4) [38]	12.0 (6.6) [43]	0.34 [−1.93 to 2.62]	0.766	
≥6: Severe avoidance, mean (SD) [n]	12.9 (6.9) [34]	14.4 (5.5) [40]	−1.33 [−3.68 to 1.02]	0.268	

Suicidal Ideation					
0: Average avoidance, mean (SD) [n]	0.3 (0.8) [23]	0.5 (0.9) [24]	−0.03 [−0.44 to 0.39]	0.900	0.444
1–2: Moderate avoidance, mean (SD) [n]	0.9 (1.1) [36]	0.6 (1.2) [30]	−0.30 [−0.67 to 0.06]	0.104	
3–5: High avoidance, mean (SD) [n]	0.9 (1.3) [35]	0.7 (1.2) [34]	0.08 [−0.26 to 0.43]	0.627	
≥6: Severe avoidance, mean (SD) [n]	1.1 (1.6) [27]	1.4 (1.7) [35]	−0.19 [−0.56 to 0.17]	0.302	

Quality of life (EQ-5D) Health index					
0: Average avoidance, mean (SD) [n]	0.7 (0.3) [24]	0.7 (0.2) [32]	−0.01 [−0.13 to 0.11]	0.851	0.524
1–2: Moderate avoidance, mean (SD) [n]	0.6 (0.3) [51]	0.7 (0.2) [34]	0.00 [−0.10 to 0.10]	0.945	
3–5: High avoidance, mean (SD) [n]	0.6 (0.3) [41]	0.6 (0.3) [47]	0.09 [−0.01 to 0.18]	0.072	
≥6: Severe avoidance, mean (SD) [n]	0.5 (0.3) [35]	0.5 (0.3) [42]	0.01 [−0.09 to 0.11]	0.813	

EQ-5D Health today					
0: Average avoidance, mean (SD) [n]	59.2 (22.6) [25]	57.5 (20.1) [32]	−2.96 [−12.64 to 6.71]	0.547	0.184
1–2: Moderate avoidance, mean (SD) [n]	58.8 (18.0) [51]	59.1 (19.0) [35]	7.73 [−0.48 to 15.95]	0.065	
3–5: High avoidance, mean (SD) [n]	52.1 (19.0) [41]	54.8 (22.1) [47]	−1.31 [−9.08 to 6.46]	0.740	
≥6: Severe avoidance, mean (SD) [n]	56.4 (20.6) [35]	49.4 (20.5) [42]	7.02 [−1.21 to 15.25]	0.094	

Recovering Quality of Life (ReQoL-20)					
0: Average avoidance, mean (SD) [n]	40.4 (13.0) [24]	43.4 (11.8) [31]	−2.97 [−8.45 to 2.50]	0.286	0.023
1–2: Moderate avoidance, mean (SD) [n]	39.5 (11.2) [49]	45.0 (13.6) [35]	−1.96 [−6.57 to 2.66]	0.405	
3–5: High avoidance, mean (SD) [n]	35.4 (12.4) [40]	40.1 (15.0) [42]	0.51 [−4.05 to 5.08]	0.825	
≥6: Severe avoidance, mean (SD) [n]	37.5 (18.7) [35]	30.6 (13.2) [39]	6.90 [2.20 to 11.60]	0.004	

Questionnaire about the Process of Recovery (QPR)					
0: Average avoidance, mean (SD) [n]	35.1 (10.2) [26]	33.5 (8.4) [32]	1.49 [−2.83 to 5.82]	0.497	0.819
1–2: Moderate avoidance, mean (SD) [n]	34.0 (9.0) [54]	34.9 (8.9) [35]	1.84 [−1.82 to 5.49]	0.323	
3–5: High avoidance, mean (SD) [n]	30.6 (11.6) [43]	31.4 (12.5) [48]	2.35 [−1.13 to 5.83]	0.184	
≥6: Severe avoidance, mean (SD) [n]	30.2 (13.8) [35]	25.6 (11.7) [44]	3.95 [0.23 to 7.68]	0.038	

a VR+TAU vs. TAU: Linear regression model for the primary outcome; modelled against treatment group, outcome score at baseline, stratification factors (site and service type) and an interaction between randomised group and the subgroup variable.

**Table 4:** Treatment effects at follow-up (26 weeks) by agoraphobic severity group.

	VR + TAU	TAU	Adjusted mean difference [95 % CI]^a^	P value for mean difference	Test of Interaction (P value)
	(N = 173)	(N = 172)			
Oxford Agoraphobic Avoidance Scale (O-AS) – avoidance score					
0: Average avoidance, mean (SD) [n]	0.6 (1.4) [25]	0.6 (1.3) [31]	−0.00 [−1.02 to 1.01]	1.000	<0.001
1–2: Moderate avoidance, mean (SD) [n]	1.3 (1.6) [50]	0.9 (1.3) [38]	0.10 [−0.73 to 0.93]	0.812	
3–5: High avoidance, mean (SD) [n]	2.7 (2.1) [45]	2.4 (2.0) [48]	0.33 [−0.45 to 1.12]	0.402	
≥6: Severe avoidance, mean (SD) [n]	3.2 (3.1) [36]	5.4 (2.1) [42]	−2.06 [−2.91 to −1.20]	<0.001	

Oxford Agoraphobic Avoidance Scale (O-AS) – distress score					
0: Average avoidance, mean (SD) [n]	31.3 (22.4) [24]	30.1 (19.6) [31]	−0.92 [−10.06 to 8.22]	0.843	0.028
1–2: Moderate avoidance, mean (SD) [n]	36.7 (17.9) [50]	33.6 (21.1) [39]	−1.03 [−8.33 to 6.27]	0.781	
3–5: High avoidance, mean (SD) [n]	46.2 (17.9) [45]	45.5 (17.2) [48]	2.03 [−4.95 to 9.01]	0.568	
≥6: Severe avoidance, mean (SD) [n]	45.5 (23.4) [36]	61.2 (15.1) [43]	−12.97 [−20.55 to −5.40]	0.001	

Agoraphobia Mobility Inventory (AMI-A)					
0: Average avoidance, mean (SD) [n]	2.3 (0.8) [22]	2.5 (0.8) [30]	−0.10 [−0.45 to 0.26]	0.594	0.004
1–2: Moderate avoidance, mean (SD) [n]	2.9 (0.6) [50]	2.5 (0.8) [36]	0.09 [−0.19 to 0.37]	0.515	
3–5: High avoidance, mean (SD) [n]	3.1 (0.5) [39]	2.9 (0.8) [44]	0.05 [−0.23 to 0.33]	0.728	
≥6: Severe avoidance, mean (SD) [n]	3.2 (1.0) [34]	3.9 (0.6) [35]	−0.61 [−0.91 to −0.31]	<0.001	

Ideas of reference (R-GPTS Part A)					
0: Average avoidance, mean (SD) [n]	7.6 (7.1) [19]	8.7 (8.3) [29]	−0.33 [−4.64 to 3.98]	0.880	0.523
1–2: Moderate avoidance, mean (SD) [n]	11.4 (8.7) [45]	9.3 (8.6) [35]	0.37 [−3.01 to 3.75]	0.829	
3–5: High avoidance, mean (SD) [n]	11.4 (10.4) [34]	10.4 (8.9) [38]	0.48 [−3.08 to 4.03]	0.792	
≥6: Severe avoidance, mean (SD) [n]	11.9 (11.3) [34]	13.1 (10.1) [32]	−2.90 [−6.53 to 0.74]	0.118	

Persecutory ideation (R-GPTS Part B)					
0: Average avoidance, mean (SD) [n]	10.3 (10.5) [19]	8.9 (10.5) [29]	0.70 [−4.83 to 6.22]	0.804	0.185
1–2: Moderate avoidance, mean (SD) [n]	13.1 (12.2) [45]	8.4 (9.6) [35]	0.72 [−3.63 to 5.07]	0.745	
3–5: High avoidance, mean (SD) [n]	13.9 (13.4) [34]	12.9 (12.7) [38]	1.53 [−3.02 to 6.09]	0.508	
≥6: Severe avoidance, mean (SD) [n]	12.9 (13.8) [34]	16.4 (15.5) [32]	−4.98 [−9.63 to −0.32]	0.036	

Overall paranoia (R-GPTS total)					
0: Average avoidance, mean (SD) [n]	17.9 (16.4) [19]	17.6 (17.7) [29]	0.40 [−8.74 to 9.54]	0.931	0.246
1–2: Moderate avoidance, mean (SD) [n]	24.5 (19.9) [45]	17.7 (17.2) [35]	0.98 [−6.20 to 8.16]	0.788	
3–5: High avoidance, mean (SD) [n]	25.4 (23.0) [34]	23.3 (19.7) [38]	1.97 [−5.56 to 9.51]	0.606	
≥6: Severe avoidance, mean (SD) [n]	24.8 (24.3) [34]	29.5 (24.8) [32]	−7.96 [−15.67 to −0.26]	0.043	

Paranoia Worries Questionnaire (PWQ)					
0: Average avoidance, mean (SD) [n]	5.7 (5.7) [19]	5.8 (5.3) [29]	−0.22 [−3.32 to 2.89]	0.890	0.219
1–2: Moderate avoidance, mean (SD) [n]	7.0 (5.7) [42]	5.2 (6.0) [35]	0.53 [−1.94 to 3.00]	0.672	
3–5: High avoidance, mean (SD) [n]	8.3 (6.5) [33]	7.8 (6.7) [40]	0.95 [−1.55 to 3.44]	0.455	
≥6: Severe avoidance, mean (SD) [n]	7.5 (6.7) [32]	9.6 (7.2) [30]	−2.64 [−5.29 to 0.00]	0.050	

Depression (Patient Health Questionnaire-9)					
0: Average avoidance, mean (SD) [n]	11.8 (7.2) [19]	10.5 (6.1) [30]	1.25 [−2.15 to 4.64]	0.470	0.295
1–2: Moderate avoidance, mean (SD) [n]	12.0 (6.1) [46]	9.5 (6.1) [35]	0.80 [−1.85 to 3.46]	0.552	
3–5: High avoidance, mean (SD) [n]	13.2 (6.3) [35]	11.2 (6.7) [38]	0.83 [−1.89 to 3.56]	0.548	
≥6: Severe avoidance, mean (SD) [n]	12.8 (7.9) [33]	15.1 (6.5) [34]	−2.27 [−5.08 to 0.55]	0.115	

Suicidal Ideation					
0: Average avoidance, mean (SD) [n]	0.3 (0.8) [22]	0.5 (1.1) [24]	−0.02 [−0.46 to 0.43]	0.944	0.723
1–2: Moderate avoidance, mean (SD) [n]	0.8 (1.1) [31]	0.5 (1.1) [30]	−0.18 [−0.57 to 0.22]	0.384	
3–5: High avoidance, mean (SD) [n]	0.9 (1.4) [32]	0.5 (1.0) [30]	0.13 [−0.25 to 0.52]	0.493	
≥6: Severe avoidance, mean (SD) [n]	1.0 (1.7) [25]	1.1 (1.7) [29]	−0.09 [−0.50 to 0.32]	0.658	

Quality of life (EQ-5D) Health index					
0: Average avoidance, mean (SD) [n]	0.6 (0.3) [22]	0.6 (0.2) [30]	−0.05 [−0.17 to 0.08]	0.462	0.314
1–2: Moderate avoidance, mean (SD) [n]	0.6 (0.3) [48]	0.7 (0.3) [36]	−0.07 [−0.16 to 0.03]	0.189	
3–5: High avoidance, mean (SD) [n]	0.6 (0.3) [38]	0.6 (0.3) [42]	0.04 [−0.06 to 0.14]	0.445	
≥6: Severe avoidance, mean (SD) [n]	0.5 (0.3) [33]	0.4 (0.3) [37]	0.05 [−0.06 to 0.15]	0.377	

EQ-5D Health today					
0: Average avoidance, mean (SD) [n]	55.5 (26.4) [22]	56.7 (20.6) [30]	−6.76 [−18.04 to 4.52]	0.239	0.442
1–2: Moderate avoidance, mean (SD) [n]	60.3 (17.8) [49]	65.1 (16.6) [36]	0.20 [−8.82 to 9.22]	0.966	
3–5: High avoidance, mean (SD) [n]	53.9 (20.9) [39]	57.0 (24.7) [42]	−2.39 [−11.38 to 6.59]	0.600	
≥6: Severe avoidance, mean (SD) [n]	52.8 (22.8) [34]	48.3 (23.7) [38]	4.96 [−4.42 to 14.34]	0.299	

Recovering Quality of Life (ReQoL-20)					
0: Average avoidance, mean (SD) [n]	43.9 (15.8) [19]	45.3 (15.2) [29]	−3.00 [−10.27 to 4.28]	0.418	0.104
1–2: Moderate avoidance, mean (SD) [n]	40.6 (13.2) [47]	46.0 (15.1) [36]	−1.95 [−7.56 to 3.66]	0.494	
3–5: High avoidance, mean (SD) [n]	36.7 (14.0) [35]	40.7 (14.0) [39]	−0.65 [−6.51 to 5.20]	0.827	
≥6: Severe avoidance, mean (SD) [n]	38.1 (18.0) [34]	31.3 (12.5) [33]	6.96 [0.94 to 12.99]	0.024	

Questionnaire about the Process of Recovery (QPR)					
0: Average avoidance, mean (SD) [n]	37.1 (10.5) [23]	36.1 (8.3) [30]	1.10 [−4.04 to 6.24]	0.674	0.626
1–2: Moderate avoidance, mean (SD) [n]	33.9 (8.8) [50]	36.4 (9.1) [37]	0.47 [−3.63 to 4.57]	0.821	
3–5: High avoidance, mean (SD) [n]	31.7 (12.8) [40]	33.1 (12.6) [45]	0.57 [−3.48 to 4.63]	0.781	
≥6: Severe avoidance, mean (SD) [n]	30.6 (14.4) [34]	25.6 (13.8) [39]	4.02 [−0.35 to 8.39]	0.071	

a VR+TAU vs. TAU: Linear regression model for the primary outcome; modelled against treatment group, outcome score at baseline, stratification factors (site and service type) and an interaction between randomised group and the subgroup variable.

## 4 Discussion

Agoraphobic avoidance is likely to prove a clinically helpful framework to view the withdrawal of many patients with psychosis. A range of fears and protective responses have been shown to be associated with agoraphobia in patients with psychosis (Rosebrock et al., 2022). Agoraphobia may be a final common result of numerous different fears. In this study a consistent pattern appears: patients showing severe agoraphobic avoidance have the highest levels of psychotic and affective symptoms. Patients with severe avoidance are doing the least and have the lowest quality of life and perceptions of recovery. This group have the highest rates of being unemployed. The Oxford Agoraphobic Avoidance Scale provides a brief assessment that identifies patients with such significant difficulties. It is highly plausible that paranoia, voices, fears, and depression lead to a tendency to withdraw, but that the withdrawal then maintains and worsens symptoms. Agoraphobia is a marker of greater clinical need in patients with psychosis. Its study and treatment need much greater attention in psychosis and also across many other mental health disorders.

It is important to highlight that the trial indicates that severe agoraphobia in patients with psychosis is treatable. There were large effect size reductions at six months. After a six-session intervention, patients on average were able to complete two more meaningful activities. Patients can overcome anxieties and return to everyday situations, even with a very brief therapy. As a patient in a qualitative study of gameChange described (Bond et al., in press): “The everyday situations that I found difficult, the more I practised them in the VR, the more I could get confident and be more confident in day-to-day life. I think because I was learning about it in the VR and practising and practising and practising, I could then take that and build up more confidence and do it in the everyday real world.” This provides great optimism for clinical services providing care to patients with severe difficulties. In effect, for patients who clinicians often struggle to help leave the home, VR provides a way to bring the outside into the home. As VR headsets become cheaper and simpler to use and therefore more easily provided to patients in their homes, individuals will be able to spend much longer with the treatment and thereby potentially improve outcomes further. It is notable that the VR therapy also brought significant improvements in paranoia and quality of life for patients with severe agoraphobia. The world was beginning to open up for these patients as they realised their fears were less realistic.

A peer-led qualitative evaluation of gameChange with 20 participants, analysed without knowledge of the trial results, also found that those individuals who were struggling the most with agoraphobic avoidance gained the most benefits from gameChange (Bond et al., in press). The interviews captured how individuals who were housebound and low in mood were often able to do activities subsequently that they had not imagined possible at the start of the VR therapy. As a participant said: “It's very different now, I can open my curtains and look out my window, I can go out to local shops and places, I can meet up with friends and family, I can do such a lot more.” The VR therapy worked best for patients with severe avoidance because the simulations and scenarios presented matched their difficulties. Patients with severe avoidance typically found it difficult to leave the house, walk down the street, and shop locally, which were exactly the kinds of scenarios presented in gameChange. In our experience these individuals were also more likely to experience ‘breakthroughs’, leading to a virtuous cycle of a sense of achievement producing an impetus to try further activities. The VR therapy will need development for patients with moderate or high – as opposed to severe - agoraphobia, for example, the addition of new relevant scenarios. Our experience in the trial with patients with lower levels of agoraphobia, who could already largely tolerate being around other people, is that the scenarios often did not trigger sufficient anxiety. This may require VR simulations of more complex social interactions. To our knowledge agoraphobia has been an outcome (and that only secondary) in just one previous randomised controlled trial for patients diagnosed with psychosis. In the GOALS trial with 75 patients with psychosis who wanted to be more active but for whom anxiety or depression was a barrier, eight sessions with a mental health staff member using a manualised therapy based on graded exposure and behavioural activation did not produce significant changes in agoraphobia (Waller et al., 2018). Treatment of agoraphobia in patients with psychosis has not been a well-studied topic and work is needed to achieve benefits for patients with a range of severity.

There are a number of limitations with the current study. The associations between agoraphobic severity levels and other difficulties cannot establish any causal relationship. The associations could simply be a result of an unmeasured confounding variable. The outcome results do demonstrate a causal relationship: that the intervention produces multiple benefits for patients with severe avoidance. However, these tests are all post hoc. The interaction term for a number of the tests was not always significant, indicating that the gains in the severe group were not always substantially greater than in the other groups. The number of participants with severe agoraphobia was fairly small and there was multiple hypothesis testing that could have led to false positive results. Replication of results would be beneficial. It is also plausible that gameChange could be helpful for patients on inpatient wards in speeding recovery and preparing for discharge (Brown et al., 2022). Following this initial work highlighting the issue, we expect future research studies and clinical trials will focus too on this potentially important clinical presentation of severe anxious avoidance in patients with psychosis.

## CRediT authorship contribution statement

DF conceived the project, design of the treatment and trial, and wrote the paper. UG, L-MY, and DF conducted the statistical analyses. FW, DMC, L-MY, and TK contributed to the design of the trial. SL, TK, FW, JF, & DMC contributed to the design of the therapy. TK led patient involvement. AR contributed to the programming of the treatment. AP, SL, LR, CA, JJ, EM, and RP co-ordinated the trial. KC, RD, AM, EO’R, FW, and DF led trial sites. JGr and AP led the data management processes. UG, L-MY, and DF had full access to all the data in this study and take responsibility for the integrity of the data and the accuracy of the data analysis. All authors commented on the paper.

## Declaration of competing interest

DF is a founder and a non-executive director of Oxford VR, a University of Oxford spin-out company, which is commercialising the gameChange treatment. DF holds equity in Oxford VR and receives personal payments. DF holds a contract for his university team to advise Oxford VR on treatment development. SL reports consultancy work and fees from Oxford VR. The University of Oxford, Oxford Health NHS Foundation Trust, and the McPin Foundation, received a share of the licencing fee from Oxford VR for the gameChange software.
